# Recent Progress in Antibody Epitope Prediction

**DOI:** 10.3390/antib12030052

**Published:** 2023-08-08

**Authors:** Xincheng Zeng, Ganggang Bai, Chuance Sun, Buyong Ma

**Affiliations:** 1 Engineering Research Center of Cell & Therapeutic Antibody (MOE), School of Pharmacy, Shanghai Jiao Tong University, Shanghai 200240, China; zeng_xincheng@163.com (X.Z.); sunchuance123@sjtu.edu.cn (C.S.); 2 Shanghai Digiwiser Biological, Inc., Shanghai 200131, China

**Keywords:** antibody, artificial intelligence, antibody design, epitope

## Abstract

Recent progress in epitope prediction has shown promising results in the development of vaccines and therapeutics against various diseases. However, the overall accuracy and success rate need to be improved greatly to gain practical application significance, especially conformational epitope prediction. In this review, we examined the general features of antibody–antigen recognition, highlighting the conformation selection mechanism in flexible antibody–antigen binding. We recently highlighted the success and warning signs of antibody epitope predictions, including linear and conformation epitope predictions. While deep learning-based models gradually outperform traditional feature-based machine learning, sequence and structure features still provide insight into antibody–antigen recognition problems.

## 1. Introduction

Monoclonal antibodies (mAbs), including antibody-based therapeutic modalities, such as bispecific antibodies [1,2], antibody–drug conjugates (ADC) [3,4], and chimeric antigen receptors (CARs) [5,6], are the most important biological drugs that are widely used to treat infectious diseases, autoimmune diseases, and cancer [7,8,9]. The antibody monomer consists of two light and two heavy chains. The two antigen-binding fragments (Fabs) recognize the specific molecular target, and the Fc region binds to immune receptors to activate effector actions. Both the light and heavy chains have three complementarity-determining regions (CDR) loops (paratopes) that bind to the antigen interface. An epitope, also known as an antigenic determinant, is a specific region on the surface of an antigen that is recognized and bound by an antibody. Epitopes can be linear or conformational, meaning that they can either be a linear sequence of amino acids or a three-dimensional (3D) structure formed by the folding of the protein. Around 10% of B-cell epitopes are linear, while the rest are non-contiguous sequences and conformational [10]. When the 3D structure of the antibody–antigen complex is available, the interactions between the paratope and epitope can be mapped and characterized well. Otherwise, as in many cases, epitopes need to be inferred or predicted by computational or experimental approaches. The characterization and prediction of antigen epitopes are not only important when designing therapeutic or diagnostic antibodies, but they are also crucial in the development of vaccines, as epitopes allow the immune system to recognize and respond to specific pathogens or abnormal cells.

Experimental techniques such as peptide microarrays or phage display libraries can be used to identify linear epitopes on a protein. Peptide microarrays involve synthesizing overlapping peptides which span the protein sequence before screening them with an antibody to identify the binding regions. Phage display libraries use bacteriophages that display peptide sequences on their surface, which can be screened for antibody binding. The native epitopes of different chemical species, including protein-, polysaccharide- and DNA-epitopes, can be replaced by peptide mimics (mimotopes). These mimotopes can be used in vaccines and diagnostics [11]. Protein sequencing analysis can also be used to identify potential linear epitope regions by probing regions with high hydrophilicity, surface accessibility, flexibility, and antigenicity. This approach can be time-consuming and may require expertise in the protein structure and function.

In contrast to linear epitopes, conformational epitopes are more difficult to map when a complex structure is not available. The hydrogen/deuterium exchange (HDX) experiment can be used to infer the antibody–antigen binding site; however, a significant drawback of using the HDX is that it can be confounded by ‘allosteric’ structural perturbation when the protein-binding effect is not limited to the binding site [12]. Not all amino acid patches on protein surfaces are suitable as an epitope for antibody binding. Current antibody technologies have difficulties when targeting several important drug targets, especially for membrane-related proteins such as G protein-coupled receptors and ion channels. To experimentally probe the possible antibody binding patches, Trkuljia et al. developed a protease incision-based method to first identify the peptide related to the binding surface and then use it to develop an antibody [13]. They use sequential protease digestion under controlled kinetic conditions to cut and release protease-accessible peptides, which are denoted as the protease-identified cut site (PIC). Using bioinformatics and structural modeling, the produced PICs are translated into a central point or coordinate in the linear or conformational epitope. Importantly, when correlated with possible functional information, the PIC can be binders, agonists, or antagonists. Eventually, synthetic antigens mimicking the PIC are used to produce optimized antibodies. This method has been demonstrated in antibody discoveries targeting the transient receptor potential vanilloid 1 (TRPV1) channel and KRAS-mutated cells [13].

The mappings of conformational epitopes are mainly predicted by various computational methods, such as homology modeling and docking simulations [14,15], molecular dynamic simulations, and quickly evolving machine learning approaches. The ML method can be roughly divided into three basic elements: the model, learning criteria, and optimization algorithm. Two commonly used ML models include Support Vector Machine (SVM) [16] and Random Forest (RF) [17]. SVM is a generalized linear classifier with a maximum–margin hyperplane to separate different data. RF combines multiple weak classifiers to produce a voted or average prediction. Recently, deep neural network algorithms have been heavily developed to study proteins and other biological molecules. Each neural network has its own strengths and is suitable for different tasks. Common deep learning (DL) models include the convolutional neural network (CNN, especially suitable for image processing) [18], variational autoencoder (VAE), and graph neural network (GNN). The VAE can capture the most critical factors which represent the input information, and the output content is trained to preserve the essential characteristics of the input [19]. GNN processes graph structure data that are represented by the nodes and edges of the graph [20], which is suitable for tasks such as node classification, edge information dissemination, and graph clustering.

It is still a challenging problem to predict the binding sites on the antigen (epitope) corresponding to a specific antibody. Traditional computational methods and ML methods are used either alone or in combination with experimental methods to characterize or predict antibody epitopes in the applications of antibody and vaccine development. Here, we provide a brief review of the antibody–antigen interaction and epitope-related predictions.

## 2. General Mechanism and Feature of Antibody–Antigen Recognition

After initial exposure to the antigen, antibodies evolve quickly in weeks from a precursor form to a mature one to recognize the antigen tightly and specifically. Based on the molecular type, we can classify three types of large molecular antigens: protein, nucleic acid, and carbohydrates. Anti-nuclear antibodies (ANAs) are often related to disease-associated autoantibodies, for example, in the case of a chronic autoimmune disease of systemic lupus erythematosus (SLE) [21]. Carbohydrate-specific antibodies occur broadly and are widespread either as natural antibodies or when produced by pathogen stimulations [22]. Most antigens are proteins, and antibody protein antigen recognition is often comparable with normal protein–protein interactions but with distinct features. Unlike epitopes, antibodies have different preferential amino acid usage for CDR’s paratope [23]. For example, Tyr and Ser dominate paratopes that are used to interact with antigen residues since Tyr can effectively interact with a broad range of antigen amino acids, including hydrophobic, polar, and charged sidechains [24].

Under the functional pressure to recognize diverse antigens through antigen-directed isolated pathways of maturation, initially linked antibodies can diverge to exhibit distinct recognition potential and recognize a wide range of antigens [25]. Kaur et al. compiled a coherent database of germline-linked mouse and human antibodies bound with distinct antigens. As expected, with common structural constraints in some CDRs, somatic mutations altered the geometries of individual antibodies. Molecular dynamic simulations provided an additional conformational landscape which indicated how the incoming pathogen led to further CDR conformational divergence while maintaining a similar overall backbone topology [25]. The analysis of multiple liganded and unliganded crystal structures of the near-germline anticarbohydrate antibodies S25–2 and S25–39 confirmed conformational flexibility [26] in antibody-antigen recognition, enabling their limited germline repertoire to match the overwhelming diversity of potential antigens [27]. Conformational selection has been proposed to be a common ligand–receptor interaction mechanism in addition to lock-and-key and induced fit [26,28,29,30]. The systematic study of antibodies S25–2 and S25–39 highlighted the conformational selection available as an evolved mechanism that preserves the inherited ability to recognize common pathogens but is still able to adapt to new threats [27]. The Molecular dynamics-based approach also captured a diverse conformational ensemble of the CDR-H3 loop to support a conformational selection mechanism upon antibody binding [31,32].

Efficient interactions between antigens and antibodies rely on conformational mobility and some on the disorder of their binding sites [33]. Like the CDR conformation changes, epitope structural flexibility represents fuzzy binding sites. Intrinsically disordered proteins exist in highly flexible conformational states and can be congruent T-cell and B-cell antigens [34]. This is consistent with the conformational selection mechanism and has been described as a “flexible lock—adjustable key” model. Even though the extreme disorder is not compatible with efficient antigen–antibody interactions and is not present in immune interactions [33], the antibody recognition of disordered antigens has the advantage of much more extensive contacts per epitope residue and better shape complementarity [35]. This can be demonstrated by many anti-amyloid antibodies [36,37] and a recent finding that antibodies specifically recognize structurally disordered Pro/Ala-rich sequences (PAS) [38]. In certain cases, protein flexibility is shown to outperform the solvent-accessible surface area as an epitope discovery metric, as illustrated in the first protein flexibility-based algorithm and its application in the Zika virus’ conserved epitope characterization [39].

Though it is not typically the primary method used for epitope prediction, MD simulations were frequently used to study the mechanism and feature of antibody–antigen recognition. Jun Zhao et al. [40] explored the recognition of monomeric, oligomeric, and fibril forms of amyloid-β (Aβ) by three homologous antibodies, namely solanezumab, crenezumab, and creneFab. Through a combination of homology modeling, molecular docking, and molecular dynamic simulations, stable complexes of antibodies with Aβ were successfully identified. The investigation revealed distinct epitopes of Aβ when interacting with each antibody. Mateusz Sikora et al. [41] conducted extensive multi-microsecond molecular dynamic simulations of fully glycosylated and palmitoylated S proteins to unveil potential antibody binding sites. Employing steric accessibility, structural rigidity, sequence conservation, and generic antibody binding signatures, this study successfully identified and established epitopes on S and introduced novel epitope candidates for a structure-based vaccine and antibody design. Luca Mollica et al. [42] employed molecular dynamic (MD) simulations in conjunction with NMR and X-ray crystallography data gathered on the wild-type HuPrP to investigate the conformational states that are present in disordered epitopes prior to Nb484 binding. This study provides valuable insights into the immunotherapeutic potential of antibodies for targeting the aggregation of flexible proteins.

New experimental assays have been developed to detect and verify antibody epitopes. For example, VirScan is an application of the phage immunoprecipitation sequencing (PhIP-Seq) method for profiling the specificities of human antiviral antibodies [43]. VirScan, and more generally PhIP-Seq, are technologies that enable high-throughput antibody analysis by combining high-throughput DNA oligonucleotide synthesis and phage display with next-generation sequencing.

Phage display can also be integrated with computational approaches to address the needs of the large-scale mapping of antigens and epitopes. For example, antibody binding epitope mapping (AbMap) can determine phage-displayed peptides bound by 202 antibodies in a single test, which are suitable for both linear and conformational epitopes [44]. An integrated platform for genome phage display (gPhage) used libraries produced from genetic material (cDNA or genomic DNA) and isolated from an organism instead of the random peptide library to represent possible linear or conformational epitopes [45]. In the case of using serum samples from patients with Chagas disease to build unbiased libraries of the eukaryotic parasite Trypanosoma cruzi, a total of 30,430 unique phage inserts encoding T. cruzi-derived antigens were identified and analyzed using bioinformatics methods to bin and cluster the possible peptides representing epitopes. The identified epitopes were further validated and complemented by online searches of the Immune Epitope Database and Analysis Resource (IEDB; www.iedb.org, acessed on 7 August 2023) for the simultaneous identification of epitopes [45].

## 3. Linear Epitope Prediction

There are two types of antigen epitope prediction methods: one with the presence of antibodies and another without. Predictions with antibodies can be used to find the most probable epitopes of the antigen, while the second group of methods can be used to identify the epitope that a known antibody binds to. Early epitope prediction methods used propensity scales to search contiguous epitope residues as long as hundred linear epitopes. Such methods include BcePred [46], ABCPred [47], and iBCE-EL [48]. BcePred used 1029 non-redundant B cell epitopes (obtained from the Bcipep database) and 1029 non-epitopes (randomly selected from SWISS-PROT database). Each physicochemical property scale consisted of 20 values, which were assigned to each of the amino acid types on the basis of their relative propensity. Prediction is based on the normalization score, which measures an average of seven maximum/minimum values from the physicochemical scale and is divided by the difference between the maximum and minimum scores. The prediction using individuals (for example, hydrophobicity, surface area, flexibility, and polarity) and their combinations generated an accuracy that was barely between 50 and 60% [46]. The BcePred was refined to ABCpred using (1) a reduced clean dataset of 700 B-cell epitopes (non-redundant, from Bcipep database) and 700 non-epitopes (randomly selected Swiss-Prot database), and (2) recurrent neural network with a single hidden layer of 35 hidden units with different peptide lengths. The new approach increased the accuracy to 65.93%.

The low performance of the individual method could be corrected using combined ensemble models (or meta-models). The iBCE-EL is an ensemble method that combines extremely randomized tree and gradient-boosting algorithms to predict the class and probability values of a given peptide. Its input features are a combination of amino acid composition and physicochemical properties. Its major features include the amino acid composition (AAC), amino acid index (AAI) [49], chain-transition-distribution (CTD), DPC, the physicochemical properties of amino acids (PCP), and various combinations of individual compositions. Using a non-redundant dataset of 5550 experimentally validated BCEs and 6893 non-BCEs from the Immune Epitope Database, six different ML algorithms (including SVM, RF, ERT, GB, AB, and k-NN) were used to select appropriate features. Finally, a combination of the above prediction models made the final prediction as commonly used in the ensemble model (EM), which performed better than individual classifiers [48].

Recently, with the development of deep neural networks, sequence-only approaches have been increasingly used in protein property predictions. When trained with the IEDB Linear Epitope Dataset, EpiDope used the peptide sequence as an input and trained a deep neural network for linear B-cell epitope prediction [50] with a ROC of 0.67, which is among the top results using several other methods. The ‘IEDB Linear Epitope Dataset’ has 1798 proteins and represents a large pathogen variety. Usually, each protein family has distinct epitope features [51]. The 1798 proteins contain 30,556 marked protein sequences, which is much larger than the 5550 sequences used in the iBCE-EL study. The improvement in EpiDope’s performance could be due to the increasing dataset and the better deep neural network architecture used (for example, bi-directional long short-term memory network (LSTM)). Indeed, a recent study using a dataset of 62,730 known linear B cell epitope sequences showed that the sequence BLAST-based method could be used to predict linear B cell epitopes. Any peptide can be considered a B cell epitope if producing ungapped BLAST hits this database with an identity ≥ 80% and length ≥ 8. Interestingly, the BLAST-based approach obtained values for the accuracy, specificity, and sensitivity of 72.54 ± 0.27%, 81.59 ± 0.37%, and 63.49 ± 0.43%, respectively [52].

## 4. Conformational Epitope Prediction

Nearly any antigen surface accessible region recognized by an antibody can be epitopes [53]. One unique cysteine scan offers better sensitivity than an alanine scan to determine conformational epitopes. Najar et al. replaced all surface residues of CcdB, a 101 residue, with a homodimeric bacterial toxin. The cysteine mutants expressed on the yeast’s surface were labeled by biotin-PEG2-maleimide. Subsequently, antibodies were screened by fluorescence-activated cell sorting (FACS) for the loss of binding to the displayed labeled mutant proteins [54]. This kind of epitope mutational analysis is better than peptide screen methods since peptide conformations are often flexible and differ from their real conformation on the folded protein surface.

Traditional computational methods tend to find specific structural features that can be used to distinguish epitope residues. Ferdous et al. studied 488 B-cell epitope structures and identified 1282 regions and 1018 fragments. Very few eiptopes (14%) contain only one region, and only 4% are truly linear, while 90% of epitopes have five or fewer regions and five or fewer fragments [55]. While some conformational epitope information can be obtained from sequence information, the accuracy is not high [56]. Conformational epitope prediction methods are usually trained with antibody–antigen structures and then characterize antigen structures using traditional geometric features, such as the number of neighbors. DiscoTope [57] defines the epitope propensity scale by a weighted sum of the contact number and the average of nearby residues’ epitope log-odds ratios. PEPITO [58] combines half-sphere exposure values at multiple distances and amino-acid propensity scores to differentiate epitope and non-epitope residues, with a performance of 75.4 AUC on the Discotope dataset. SEPPA [59] used the following procedure to analyze each antigen protein from the input:

Step 1: Determine all the surface residues in the protein antigen;

For each surface residue r:

Step 2: Search all possible unit patches within a 15 Å atom distance of residue r, map the pre-calculated propensity indices to the above unit patches, and calculate the propensity index avgr;
Step 3: Calculate the clustering coefficient (ccr) for residue r using the Equation;
Step 4: Summarize avgr and ccr as the antigenicity score for residue r;
Step 5: Give the antigenicity score for each residue and highlight those residues with scores higher than a threshold.

EpiPred [60] identifies the epitope region by a combination of the specific antibody–antigen score and the conformational matching of antibody–antigen structures. The score function uses a graph-based approach by defining the node as a possible intermolecular contact between the antibody and antigen residues. Two nodes may be connected by an edge only if the difference in their intramolecular distances on the antibody and the antigen is below 1 Å. Finally, the score is the sum of the products of a degree of node n, and the preference of two amino acids conforms to a node.

**Figure 1:**
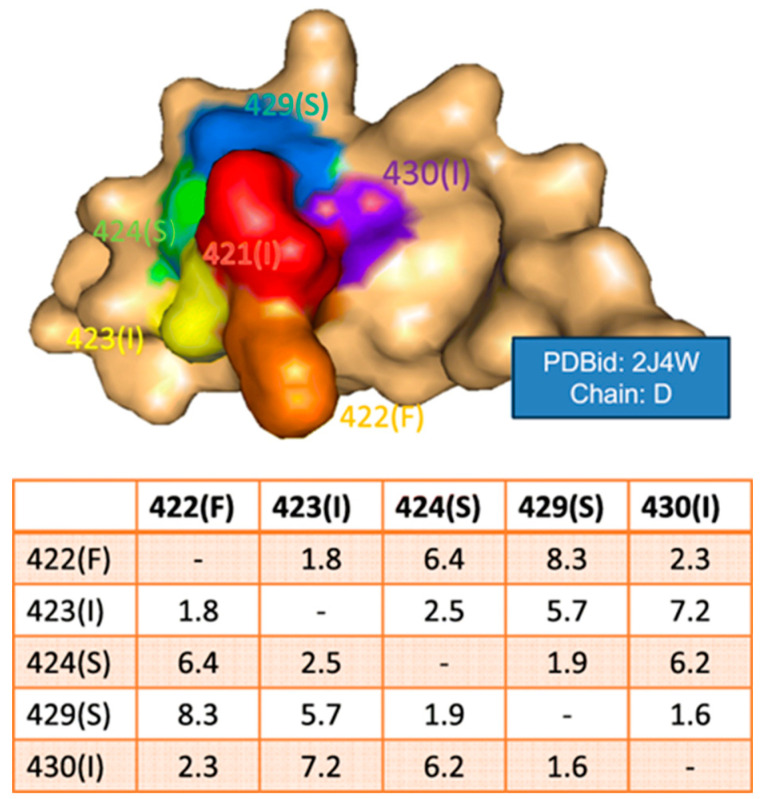
Illustration of a surface spiral vector. The table lists the shortest distance for a group of neighboring surface residues. From reference [61].

The surface spiral vector has been used to characterize conformational epitope patches [61]. The procedure to generate the surface spiral vector starts by obtaining all the adjacent residues of each surface residue first. Then, the shortest distance between all pairs of neighboring surface residues was calculated and ranked. Finally, the sequence of contact residues was obtained as the spiral feature with the shortest distance (Figure 1). Thus, sequence and surface patch matching were combined for conformational epitope prediction.

Many conformational epitope predictors have been published and are available online as web servers. Hu et al. evaluated the performance of the ensemble model (or meta-learning model) for conformational epitope prediction [62]. The base features used included the propensity score of amino acids in the spatial neighborhood, residue accessibility (all-polar, nonpolar, total-side, and main-chain), an accessible surface area, the solvent excluded surface, antigenic propensity, secondary structure, B factor, etc. They have shown that the meta-learning approach for epitope prediction integrated the complementary predictive strengths of different models, and this combined approach is much better than single epitope predictors [63]. However, Cia, Pucci, and Rooman tested nine conformational epitope predictor webservers on a dataset of over 250 antibody–antigen structures. Unfortunately, all the methods, including generic and antibody-specific methods, achieved very low performances. Commonly used consensus ensemble strategies are only marginally better than random selection. Using the SARS-CoV-2 spike protein as an independent case study largely recapitulated the benchmarking conclusions. Apparently, to improve the performance of conformational epitope prediction methods, new strategies are definitely needed [63].

With the introduction of deep learning models, conformational epitope predictions can combine the sequence and structure with local and global features to improve antibody epitope predictions. BCEs [64] extracted the antigen’s local and global features using two parallel modules. The local features were at the residues level, which were processed using Graph Convolutional Networks. The global features describe the entire antigen using all sequence information extracted with Attention-Based Bidirectional Long Short-Term Memory networks. SEMA [65] used a transfer learning approach to predict epitopes based on the primary antigen sequence and tertiary structure. The authors generated a non-redundant dataset of antigen–antibody complexes in the PDB database. The pretrained protein large language model, ESM-1v, was used to re-train the conformational epitope dataset and predict the linear epitope (SEMA-1D). Interestingly, the protein structure prediction model ESM-IF was used in parallel to quantitatively predict antibody–antigen interaction features and predict conformational epitope residues (SEMA-3D).

Epitope3D is a novel scalable machine-learning method that is capable of accurately identifying conformational epitopes when trained and evaluated on the largest curated epitope dataset to date. The method models epitope and non-epitope regions as graphs using graph-based signature concepts and extracts distance patterns as evidence for the training and testing of predictive models [66]. The results showed Epitope3D to be superior to existing alternative methods with cross-validated Mathew correlation coefficient and f1 scores of 0.55 and 0.57, respectively, and an independent blind test Mathew correlation coefficient and f1 scores of 0.45 and 0.36, respectively.

## 5. Epitope Prediction Based on Paratope–Epitope Interactions

It is still a great challenge to map and predict the paratope, epitope, and paratope–epitope interactions [67]. Due to the special sequence feature, paratope prediction is usually more accurate than epitope prediction. Similar to the methods used in conformation epitope prediction, Parapred incorporates both local residue neighborhood information and the overall sequencing information [68] of CDR without the consideration of the antigen. Using structural alignments of similar antigen–antibody complexes, Paramatome identifies consensus antigen-binding regions and uses them as a reference set of antibody–antigen complexes to identify the antibody-binding regions [69]. Using self-attention convolutions, AG-Fast-Parapred [70] significantly reduces computation time and moderately improves accuracy (AUC = 0.90) compared to Parapred (AUC = 0.88).

Using the antigen sequence and structural features only may not provide enough information to predict the antibody epitope. It is natural to hope that a combination of known paratope–epitope pairing features can boost accuracy. While the antibody–antigen interaction prediction could be more complex than only predicting the epitope, the correct prediction of association between the paratope and epitope implies predicting the epitope correctly. Indeed, a unified DL-based antibody–antigen predictor PECAN predicted epitopes by the paratope prediction networks, which was better than the networks trained solely for epitope prediction [71]. It uses transfer learning. A base graph convolutions network trained on general proteins is used as the initialization for training the epitope and paratope prediction networks. In the graph representation of protein, the amino acid residues are nodes and edges connected to residues with a Cβ–Cβ distance less than 10 Å. Nodes in the antibody graph are limited to ‘CDR clouds’ by considering two sequentially adjacent CDR residues and other residues within 6 Å in the structure [71].

In addition to the CDR graph, the PECAN predicts the paratope and epitope ‘symmetrically’ since both the paratope and epitope information are trained in one model. To separately train the paratope and epitope and make use of antibody sequence information, asymmetrical training models were developed in EPMP for prediction (Para-EPMP) and epitope prediction (Epi-EPMP) predictors [72]. This method adopts separate neural message-passing architectures that are specifically designed for paratope and epitope prediction and improved in both tasks. Para-EPMP combined sequence and structural graphs as input features, while Epi-EPMP only used structural information [72].

**Figure 2:**
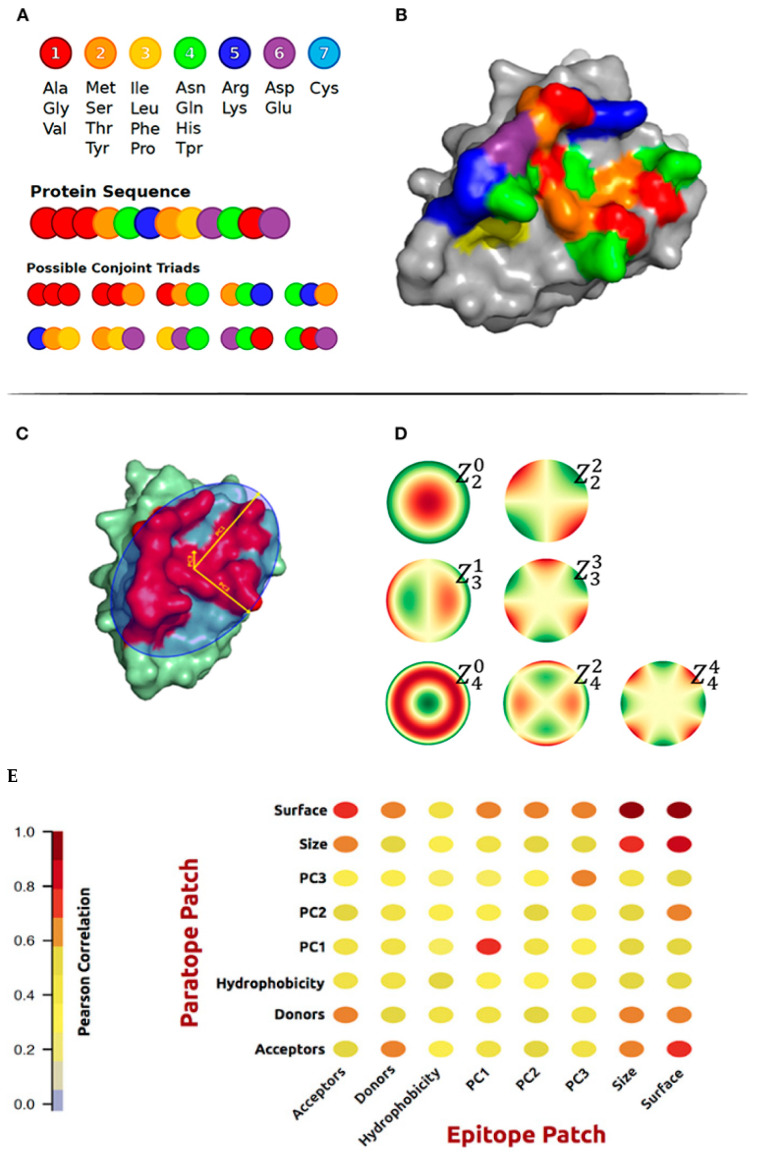
(**A**) Representation of the method and definition of Conjoint Triad amino acid classes on a sequence level. (**B**) Conjoint Triad classes mapped to an epitope patch structure. (**C**) Illustration of the three principal components on an epitope patch. (**D**) The 4th order of Zernike Moments’ descriptive shape excluding orders 0 and 1. (**E**) Correlation matrix of structural and physicochemical features of the true paired paratope and epitope patches. From reference [73].

Jespersen et al. studied the geometric and physicochemical features that are correlated in interacting paratopes and epitopes derived from the antigens and their cognate antibodies structures [73]. In addition to the commonly used amino acid composition and hydrophobic score, they generated and characterized conjoint triads amino acid classes and surface patches for actual epitope–paratope pairs. Amino acids were assigned to one out of seven classes (Figure 2A). The geometric features included principal components calculated on patches of x, y, z coordinates and Zernike moments. Zernike moments are an image descriptor that is used to characterize the shape of an object in an image. The shape to be described can either be a segmented binary image or the boundary of the object (Figure 2D). They investigated correlations between the physicochemical and structural properties of known paratope and epitope patches. As expected, a high correlation between the corresponding structural properties of the paratope and epitope was found (Figure 2E). Finally, these features were used to train AI models to predict epitopes.

**Figure 3:**
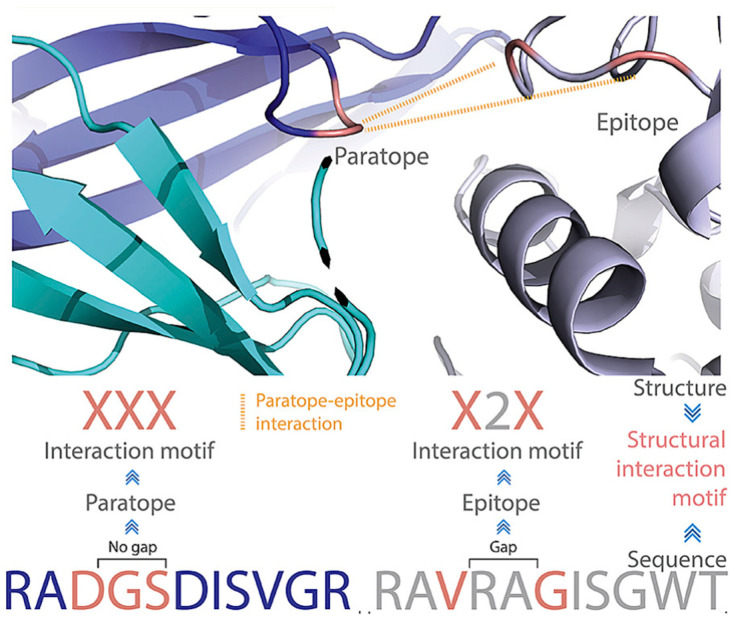
A structural interaction motif notation that accounts simultaneously for gaps and interacting residues in both paratopes and epitopes. Reproduced from reference [74].

The above examples used different descriptors for surface structural features. Akbar et al. extensively examined antibody–antigen structural interaction motifs [74]. This motif was composed of interacting paratope and epitope amino acid residues, which were encoded as capital X. The non-interacting residues (gap) were encoded as integers, which quantified the number of non-interacting amino acid residues (Figure 3). They found that using less than 104 commonly shared structure motifs, it was possible to enable the machine learnability of antibody–antigen binding on the paratope–epitope level using generative machine learning [74]. These motifs are unique for antibody–antigen recognition and are distinct from non-immune protein–protein interactions. The commonly shared motifs mediate specific oligo- and polyreactive interactions between paratope–epitope pairs. The uniqueness of these motifs is understandable since amino acid preference in mediating antibody–antigen interactions is totally different from normal protein–protein interactions.

The existence of 10^4^ commonly shared structure motifs implies polyreactive interactions. One interesting observation in antibody–antigen recognitions is that sequence-dissimilar antibodies can bind to the same epitope. Such examples include anti-lysozyme antibodies, the anti-HIV core protein gp120, and, recently, anti-COVID-19 antibodies. All these groups have dissimilar CDRH3 sequences against highly similar epitopes. Trained from 920 antibody–antigen complexes, Ab-Ligity is able to predict antibodies that could bind to highly similar epitopes (precision of 0.95 and recall of 0.69) [75]. The coding of paratope–epitope interaction pairs considers all combinations of triplets formed from a set of tokenized residues in a binding site. The edge of each triplet is represented by its vertices’ tokens and length. Each combination of tokens has a unique six-letter hash code. One may notice that Ab-Ligity shares similar features highlighted in Figure 3.

The Bepar (B-cell epitope prediction through association rules) method analyses association patterns between antibody and antigen residues that have cooperativities within the binding site, providing spatial relations within the paratope and epitope [76]. Based on the statistics of antibody–antigen complexes, Zhao and Li found that the top ten frequent association (bi-cliques: Ab-Ag) was D-K, Y-E, Y-N, S-E, Y-K, N-R, Y-R, (D,Y)-K, (S,Y)-Q, and G-R. In the meantime, they also identified co-occurrent epitope–paratope interacting residue pairs; for example, the frequent interaction residue pairs Y-K and S-Q often came with Y-Q as a co-occurrent pair. One could notice from their study that most paratope residues were Y and S, and the associated epitope residues included charged residues [76]. This trend has been confirmed in a later study that antibodies frequently use Tyr to interact with charged residues in antigen residues [24].

## 6. Using Antibody–Antigen Dock to Predict Conformational Epitope

With a similar argument to improve epitope prediction by considering the antibody paratope interaction, protein docking has been frequently used in epitope prediction and provides additional information about the overall quaternary structure of the antibody–antigen complex from their separate tertiary structures. Again, despite considerable progress in protein docking, selecting near-native models out of many structural combinations remains a challenging task [77].

Ambrosetti et al. compared four different docking methods (ClusPro, LightDock, ZDOCK, and HADDOCK) for their ability to predict antibody–antigen binding interfaces, including 16 antibody–antigen complexes [78]. When a single structure was used, all methods achieved good results for the most rigid structures when a vague definition of the epitope was provided. In the case of allowing a limited conformational change in the antibody, HADDOCK and LightDock did not achieve a striking better performance compared with rigid ClusPro or ZDOCK. When experimental information about the interface was provided, HADDOCK led to the generation of a much higher number of good models [78].

As one of the best protein–protein dock search engines, the ZDock docking algorithm was widely used in protein–protein docking, even though it was not specifically optimized for antibody–antigen docking. Therefore, it was expected to improve the ranking of ZDock and predict antibody–antigen binding, as DLAB-Re did [79]. DLAB retrained a CNN with a dataset of 1216 antibody–antigen complexes. In total, 759 non-redundant complexes were selected if their CDR sequence was only present once in the dataset. The CNN input was derived from the atom information in four-dimensional grids: three for the interaction site and one for atom types.

For antibodies or antigens without a 3D structure available, homology models can also be used in the docking procedure to predict paratopes and epitopes. As expected, studies using MAbTope [15] and homology models indicated that overall accuracies depend on the method chosen for homology modeling and the templates used [14]. For antibody modeling, the highest sequence identity often comes from the framework region, whereas antibody specificity is mostly due to CDRs. Nevertheless, they have shown that even low-quality models can be used to predict epitopes. Using this method, the epitope of an anti-IL4 receptor alpha subunit therapeutic antibody (dupilumab) of an unknown 3D structure was predicted and validated experimentally [14]. Even though the docking result could not be 100% right, large-scale docking could be combined with the experimental “epitope binning” of monoclonal antibodies using a high-throughput surface plasmon resonance to reveal which antibodies competed and why and where they might compete in terms of possible binding sites on the antigen [80]. Brooks et al. combined experimental binning with “dock binning”. This approach is useful when a group of antibodies targeting a common antigen is known. Therefore, based on homology models of all these known antibodies, docking results can be grouped for “binning” to compare with the experimental antigen screening using these antibodies. The cross-comparison/validations among experimental and computational docking results provide information on the group-level identification of functionally related monoclonal antibodies (i.e., communities) and the identification of their general binding regions on the antigen [80]. The Bailey–Kellogg group also developed a docking-based strategy to experimentally test the docking results and correlate this with epitope identification. Based on docking poses, three amino acid mutations on the putative antibody–antigen binding surface were designed and tested experimentally. In the case of positive identification, the mutations should disrupt the antibody–antigen binding, thus confirming their computational predictions [81].

With the widely available use of an accurate protein structure predictor like AlphaFold2, a better docking prediction of the epitope could be achieved compared to traditional homology modeling. AbAdapt is a pipeline that integrates AlphaFold structural modeling with antibody and antigen rigid docking in order to derive antibody-antigen-specific features for epitope prediction. Incorporating more accurate antibody models, an improvement in docking, paratope prediction, and the prediction of antibody-specific epitopes can be achieved [82].

## 7. Conclusions

We have discussed traditional and current approaches for the prediction of antibody-antigen epitopes, many of which are available online (Table 1). The prediction and identification of antibody epitopes are important for disease diagnostics, vaccine development, and the development of antibody therapy. With advances in the application of deep learning-based AI in protein science, the prediction of antibody epitopes and their interactions with the antibody are more and more accurate. The current structural-based dataset provided information on the static features of antibody–antigen binding, yet how to include conformation dynamics in flexible antibody–antigen binding remains a challenge. The increasing entries of linear epitope sequence greatly improved prediction accuracy, and prediction conformation epitope could be helped by considering paratope–epitope association patterns. Deep learning-based models gradually outperform traditional feature-based machine learning; however, sequence and structure features still provide insights into antibody–antigen recognition problems. The current structural-based dataset provides information on the static features of antibody–antigen binding, yet how to include conformation dynamics in flexible antibody–antigen binding remains a challenge.

## Figures and Tables

**Table 1:** List of epitope prediction severs discussed in this work. (all accessed on 7 August 2023).

Method Name	Year	Methodology/Approach	Link
Bcepred	2004	prediction of linear B-cell epitopes, based on physicochemical properties	http://crdd.osdd.net/raghava/bcepred
ABCpred	2006	prediction of linear B-cell epitopes, based on recurrent neural network	http://crdd.osdd.net/raghava/abcpred
iBCE-EL	2018	prediction of linear B-cell epitopes, based on a fusion of randomized tree (ERT) and gradient boosting (GB) classifiers	http://thegleelab.org/iBCE-EL
EpiDope	2021	prediction of linear B-cell epitopes, based on bi-directional long short-term memory network (LSTM)	http://github.com/mcollatz/EpiDope
PECAN	2020	prediction of B-cell epitopes by paratope–epitope interactions, based on graph Convolution Attention Network and transfer learning	https://github.com/vamships/PECAN.git
EPMP	2021	prediction of B-cell epitopes by paratope–epitope interactions, based on separate neural message passing architectures	https://arxiv.org/abs/2106.00757
Jespersen et al.	2019	prediction of B-cell epitopes by paratope–epitope specific interaction rules, based on geometric and physicochemical features, statistical and machine learning algorithms	https://doi.org/10.3389/fimmu.2019.00298
Akbar et al.	2021	prediction of B-cell epitopes by paratope–epitope interactions, based on antibody–antigen interaction motifs	https://doi.org/10.1016/j.celrep.2021.108856
